# Function-specific IL-17A and dexamethasone interactions in primary human airway epithelial cells

**DOI:** 10.1038/s41598-022-15393-2

**Published:** 2022-06-30

**Authors:** Siti Farah Rahmawati, Rémon Vos, I. Sophie T. Bos, Huib A. M. Kerstjens, Loes E. M. Kistemaker, Reinoud Gosens

**Affiliations:** 1 Department of Molecular Pharmacology, University of Groningen, Antonius Deusinglaan 1, 9713 AV Groningen, The Netherlands; 2 Department of Pharmacology and Clinical Pharmacy, Institut Teknologi Bandung, Bandung, Indonesia; 3 Department of Pulmonary Medicine, University of Groningen, University Medical Center Groningen (UMCG), Groningen, The Netherlands; 4 Aquilo Contract Research, Groningen, The Netherlands; 5 University of Groningen, University Medical Center Groningen (UMCG), Groningen Research Institute for Asthma and COPD, Groningen, The Netherlands

**Keywords:** Preclinical research, Pharmacology

## Abstract

Asthmatics have elevated levels of IL-17A compared to healthy controls. IL-17A is likely to contribute to reduced corticosteroid sensitivity of human airway epithelium. Here, we aimed to investigate the mechanistic underpinnings of this reduced sensitivity in more detail. Differentiated primary human airway epithelial cells (hAECs) were exposed to IL-17A in the absence or presence of dexamethasone. Cells were then collected for RNA sequencing analysis or used for barrier function experiments. Mucus was collected for volume measurement and basal medium for cytokine analysis. 2861 genes were differentially expressed by IL-17A (Padj < 0.05), of which the majority was not sensitive to dexamethasone (< 50% inhibition). IL-17A did inhibit canonical corticosteroid genes, such as *HSD11B2* and *FKBP5* (p < 0.05). Inflammatory and goblet cell metaplasia markers, cytokine secretion and mucus production were all induced by IL-17A, and these effects were not prevented by dexamethasone. Dexamethasone did reverse IL-17A-stimulated epithelial barrier disruption, and this was associated with gene expression changes related to cilia function and development. We conclude that IL-17A induces function-specific corticosteroid-insensitivity. Whereas inflammatory response genes and mucus production in primary hAECs in response to IL-17A were corticosteroid-insensitive, corticosteroids were able to reverse IL-17A-induced epithelial barrier disruption.

## Introduction

IL-17 is a pro-inflammatory cytokine that is central in physiological responses such as host defense regulation and tissue repair. However, it also plays a role in the pathogenesis of several diseases. For example, IL-17A plays an important role in aggravating the inflammatory processes in airway diseases such as asthma and chronic obstructive pulmonary disease (COPD)^1^. The levels of IL-17A in sputum, nasal and bronchial biopsies and serum have been reported to be higher in asthma and COPD patients than healthy controls^2–6^. Moreover, asthma severity has been linked with more elevated levels of IL-17A in these sputum, nasal and bronchial biopsies and serum^3,4,7–9^. In COPD patients, the presence of an IL-17A gene signature in airway epithelial cells was associated with increased airway obstruction and functional small airways disease, as well as reduced sensitivity to corticosteroids^10^.

The main anti-inflammatory drugs used clinically in asthma and COPD are corticosteroids^11,12^. Problematically, the effects of corticosteroids are limited in patients with severe asthma and in patients with COPD^13^. Several stimuli, such as pathogens, allergens, and cigarette smoke, as well as cytokines (IFN-γ, IL-17A, IL33, TSLP, TNFα, TGFβ) have been shown to promote corticosteroid insensitivity in severe asthma and COPD^13^. Indeed, there is a growing body of evidence for reduced corticosteroid sensitivity in response to IL-17A driven inflammation. Such corticosteroid insensitivity in response to IL-17A has been reported in immune cells, such as T helper cells and PBMCs, as well as structural cells, such as airway smooth muscle and epithelial cells^14^.

Epithelial cells have a major role in the airways by acting as barrier in the airway and by regulating immune responses^15^. In asthma and in COPD, there is a shift in epithelial differentiation leading to higher goblet cell number and exaggerated mucus production^16^. IL-17A exerts its effect on epithelial cells through binding to its receptor (IL-17R) and stimulates granulocyte-colony stimulating factor (G-CSF) and neutrophil recruiting chemokines, such as C-X-C ligand 1 (CXCL1), CXCL2, and CXCL5, causing neutrophilic inflammation^17^. Neutrophilic inflammation is known to be poorly sensitive to corticosteroids^18–20^. Moreover, in human airway epithelial cells, IL-17A pre-treatment reduced the effect of budesonide in preventing TNF-α-induced IL-8 production in vitro^21^. This indicates that IL-17A-induced insensitivity to corticosteroids might be the result of a direct effect on airway epithelial cells and might not necessarily be neutrophil-dependent. However, the functional responses via which IL-17A represses glucocorticosteroid signaling in airway epithelial cells, as well as the functional responses sensitive to this mechanism, are not yet fully clarified. Therefore, in this study, we investigated the functional and molecular interactions of IL-17A and dexamethasone, a potent corticosteroid, on primary human airway epithelial cells. We demonstrate that IL-17A induces function-specific corticosteroid insensitivity, which was evident for inflammatory responses and mucus production, whereas the effects of dexamethasone on epithelial barrier were maintained.

## Results

### Effect of IL-17A and dexamethasone on primary human airway epithelial cells

**Figure 1:**
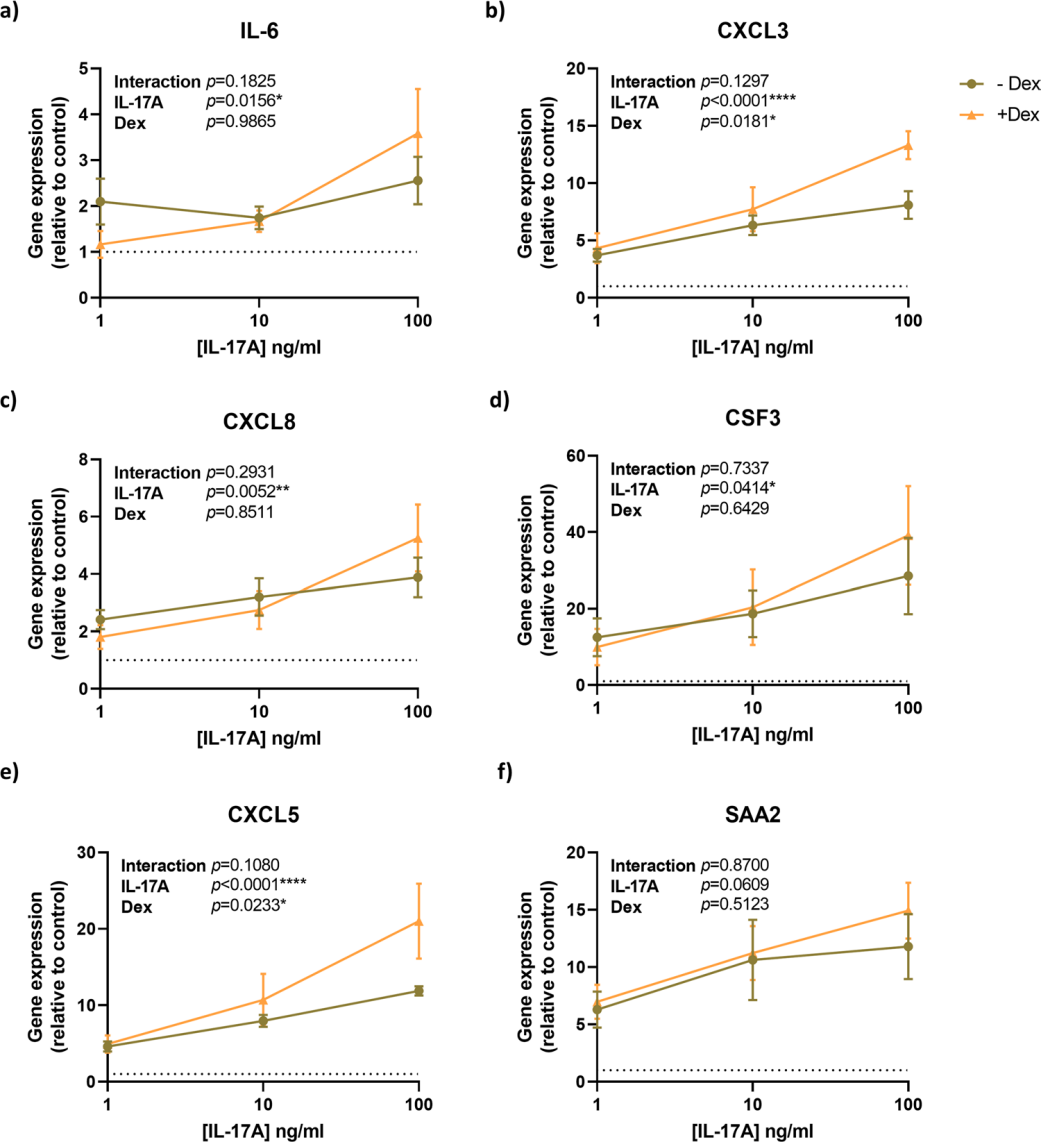
IL-17A stimulates IL-17 signature genes in basal airway epithelial cells (hAECs). hAECs were cultured submerged and exposed to IL-17A (10 ng/ml) with or without dexamethasone (Dex; 10 nM) for 24 h. Expression of (**a**) *IL-6*, (**b**) *CXCL3*, (**c**) *CXCL8*, (**d**) *CSF3*, (**e**) *CXCL5*, (**f**) *SAA2* were analyzed by real-time qPCR. Data represent means ± SEM. N = 4 donors, [IL-17A] = IL-17A concentration. Statistical analyses were performed with two-way ANOVA, *p < 0.05, **p < 0.01, ****p < 0.0001.

To validate the effect of IL-17A on primary human airway epithelial cells (hAECs), we stimulated the cells with three concentrations of IL-17A: 1, 10, and 100 ng/ml. To study corticosteroid insensitivity, cells were exposed to 10 nM dexamethasone. Markers previously described characteristic for IL-17A sensitivity (*IL6*, *CXCL8*, *CXCL3*, *CSF3*, *CXCL5*, and *SAA2*) were assessed^10^. All were indeed induced by IL-17A in dose dependent manners (Fig. 1a–f). The expression of these genes was not altered by the presence of dexamethasone. Furthermore, addition of dexamethasone tended to further induce expression of these genes in response to IL-17A 100 ng/ml stimulation. This validates the IL-17A-gene-signature sensitivity in hAECs as well as its corticosteroid insensitive nature (Fig. 1a–f). Based on these data, we chose IL-17A at a concentration of 10 ng/ml for further experiments.

### Effect of IL-17A on differentiated hAECs grown at an air liquid interface (ALI)

**Figure 2:**
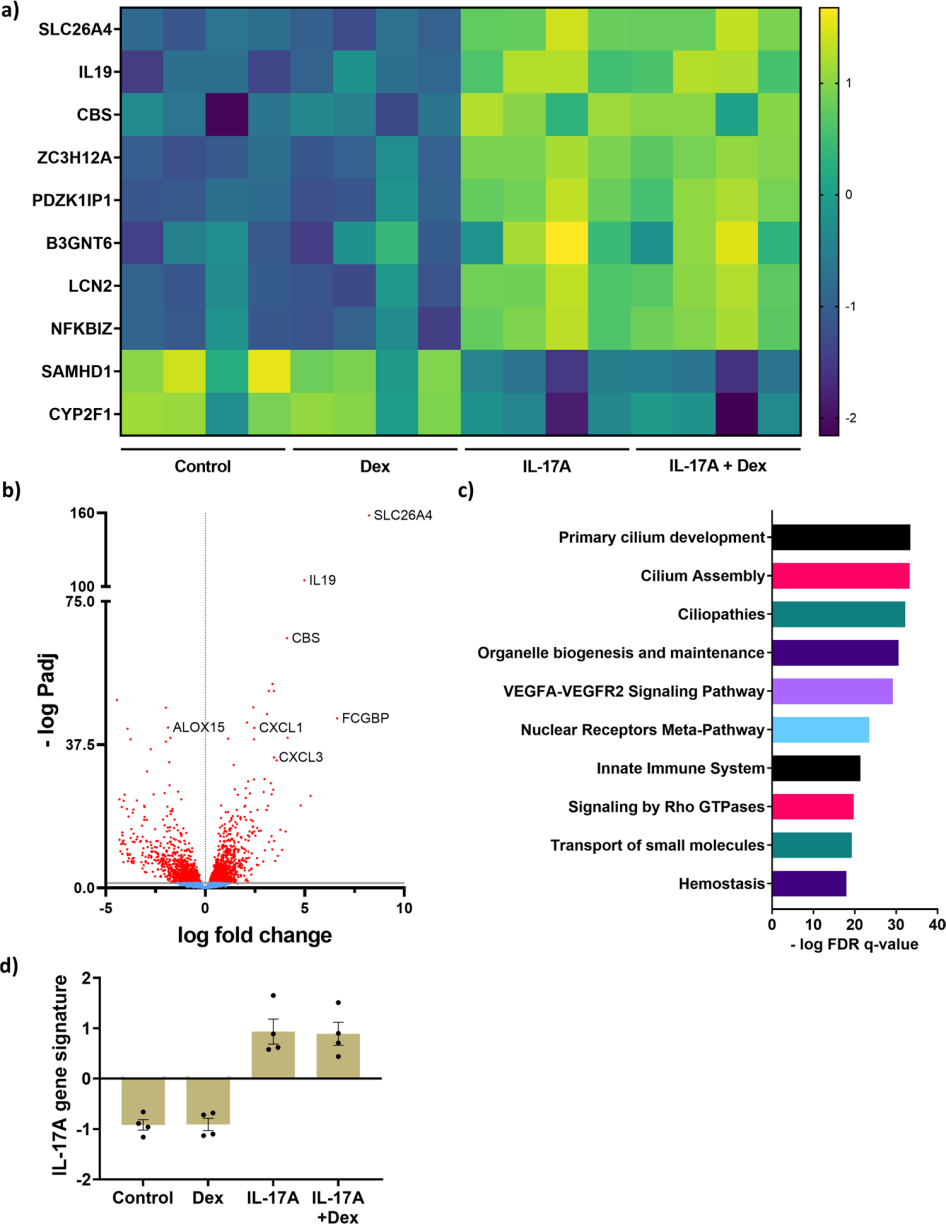
IL-17A induces gene expression that is insensitive to dexamethasone (Dex) in human airway epithelial cells (hAECs). hAECs were cultured and differentiated at an air liquid interface (ALI) and exposed to IL-17A (10 ng/ml) with or without dexamethasone (10 nM) for 14 days. Gene expression was determined by bulk RNA sequencing and differentially expressed genes were analyzed and presented as heatmap of the top 10 most regulated genes, with each column in the groups representing an individual donor (**a**) and by a volcano plot (**b**). Pathway analysis (GSEA) of the statistically significant genes was done and presented as the top 10 most regulated pathways (**c**). The IL-17A gene signature of the top 10 differentially expressed genes was calculated as Z-score ratio (**d**). Data represent means ± SEM. N = 4 donors.

Next, we characterized the effect of 10 ng/ml IL-17A and 10 nM dexamethasone in hAECs grown and differentiated at an air liquid interface (ALI) for 14 days. We exposed the cells to IL-17A, dexamethasone, or the combination during the 14 day differentiation period. The top 10 genes regulated by IL-17A were *SLC26A4, IL-19, CBS, PDZK1IP1, ZC3H12A, B3GNT6, CYP2F1, NFKBIZ, SAMHD1*, and *LCN2*, the expression of which was not inhibited by the presence of dexamethasone (Fig. 2a). 2861 genes were significantly differentially expressed by IL-17A (*Padj* < 0.05), of which 1439 were significantly upregulated and 1422 genes were significantly downregulated (Fig. 2b). Importantly, we found the same genes to be regulated by IL-17 in the air liquid interface design that we independently observed in the submerged design (Fig. 1). These genes were previously reported to be part of the IL-17A gene signature by others^10^, further supporting the findings.

We further analysed the IL-17A differentially expressed genes by gene set enrichment analysis (GSEA) using three pathway definitions: Kyoto Encyclopedia of Genes and Genomes (KEGG), reactome, and wikipathways (http://www.gsea-msigdb.org). Genes induced by IL-17A clustered into more than 100 pathways, the top 10 pathways are presented in Fig. 2c. The significantly enriched pathways in response to IL-17A included *Cilia function and development*, *Neutrophil degranulation, and Extracellular matrix organization* (FDR q-value < 0.05), which are known to be regulated by IL-17A^22^. For the top 10 genes (Fig. 2a), an IL-17A gene signature Z-score was calculated for each group. There was no difference between IL-17A with and without dexamethasone. This analysis confirmed that dexamethasone had no effect on the top 10 IL-17A gene signature (Fig. 2d).

### Effect of dexamethasone on differentiated hAECs grown at an air liquid interface (ALI)

**Figure 3:**
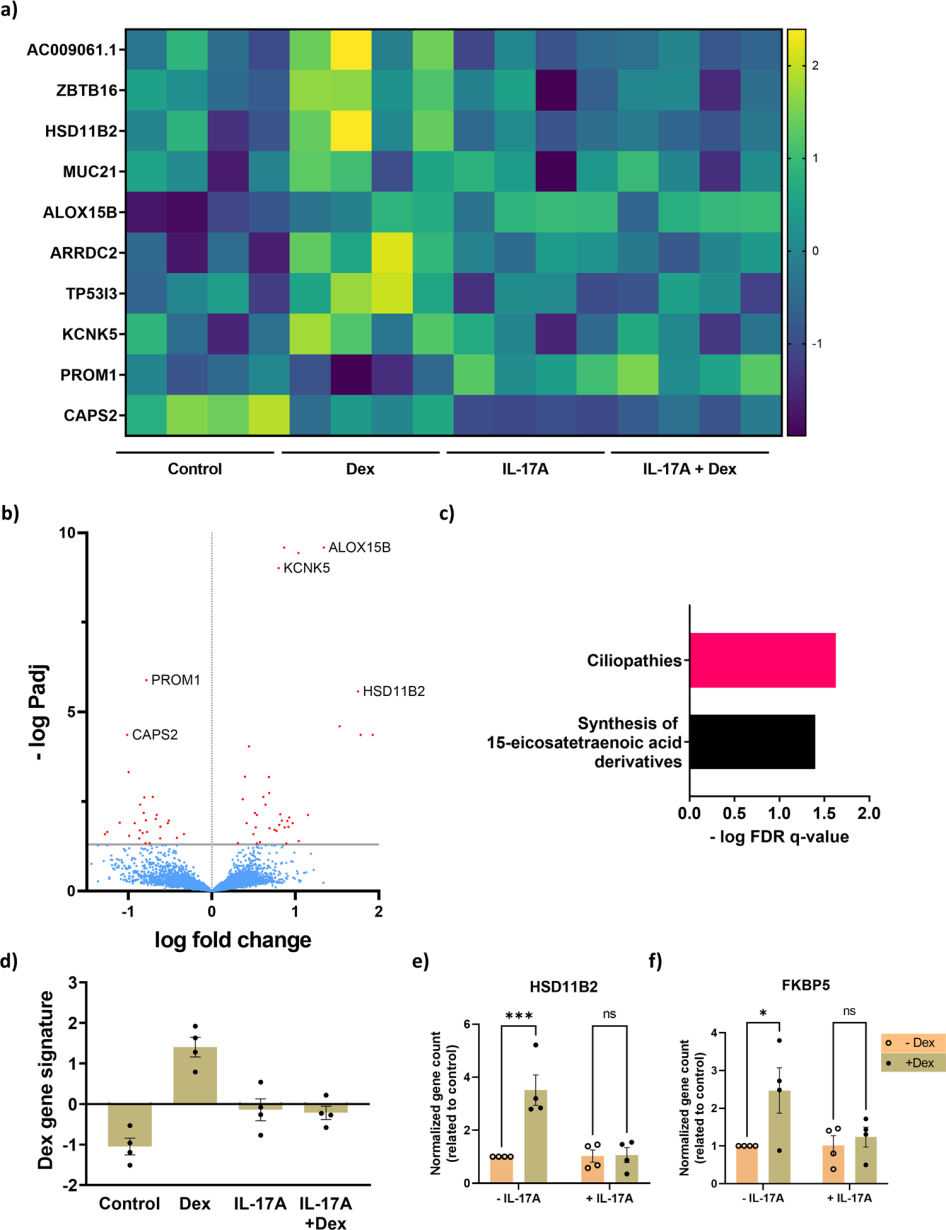
Dexamethasone (Dex)-inducible genes are dampened by the presence of IL-17A in human airway epithelial cells (hAECs). hAECs were cultured and differentiated at an air liquid interface (ALI) and exposed to IL-17A (10 ng/ml) with or dexamethasone (10 nM) for 14 days. Gene expression was determined by bulk RNA sequencing and differentially expressed genes were analyzed and presented as a heatmap of the top 10 most regulated genes by dexamethasone, with each column in the groups representing an individual donor (**a**) and by a volcano plot (**b**). Pathway analysis (GSEA) of the differentially expressed genes was done subsequently (**c**). The dexamethasone gene signature was calculated as Z-score ratio of the most regulated genes (**d**). Canonical corticosteroid gene expression, *HSD11B2* (**e**) and *FKBP5* (**f**), were analyzed from normalized gene counts. Data represent means ± SEM. N = 4 donors. Statistical analyses were performed with paired two-way ANOVA and Šidák’s multiple comparison test, *p < 0.05, ***p < 0.001.

The top 10 genes regulated by dexamethasone were *ALOX15B, TP53I3, ARRDC2, KCKNK5, PROM1, HSD11B2, MUC21, CAPS2, ZBTB16*, and *AC009061.1* (Fig. 3a). Interestingly, the presence of IL-17A prevented the regulation of these genes. 64 genes were significantly differentially expressed, of which 38 genes were significantly upregulated and 27 genes were significantly downregulated (*Padj* < 0.05) (Fig. 3b). Only two pathways were significantly regulated by dexamethasone, including cilia function and development, which were driven by *CEP41, TMEM67, ARL6, NME7, DNAL1* gene expression (FDR q-value < 0.05) (Fig. 3c). Interestingly, these genes were also significantly regulated by IL-17A alone, as previously shown in Fig. 2c. Moreover, the calculated dexamethasone signature gene Z-score was also inhibited by IL-17A (Fig. 3d). This indicates a dexamethasone effect in ciliated cell differentiation and its interaction with IL-17A in regulating this pathway.

In addition, we analyzed two canonical corticosteroid genes that were reported in literature, *HSD11B2* and *FKBP5*. *HSD11B2* and *FKBP5* gene expression was induced by dexamethasone, which was prevented almost completely by IL-17A (Fig. 3e,f)^23,24^. Together, the results indicate that IL-17A inhibits dexamethasone signaling.

### Interactions between IL-17A and dexamethasone

**Figure 4:**
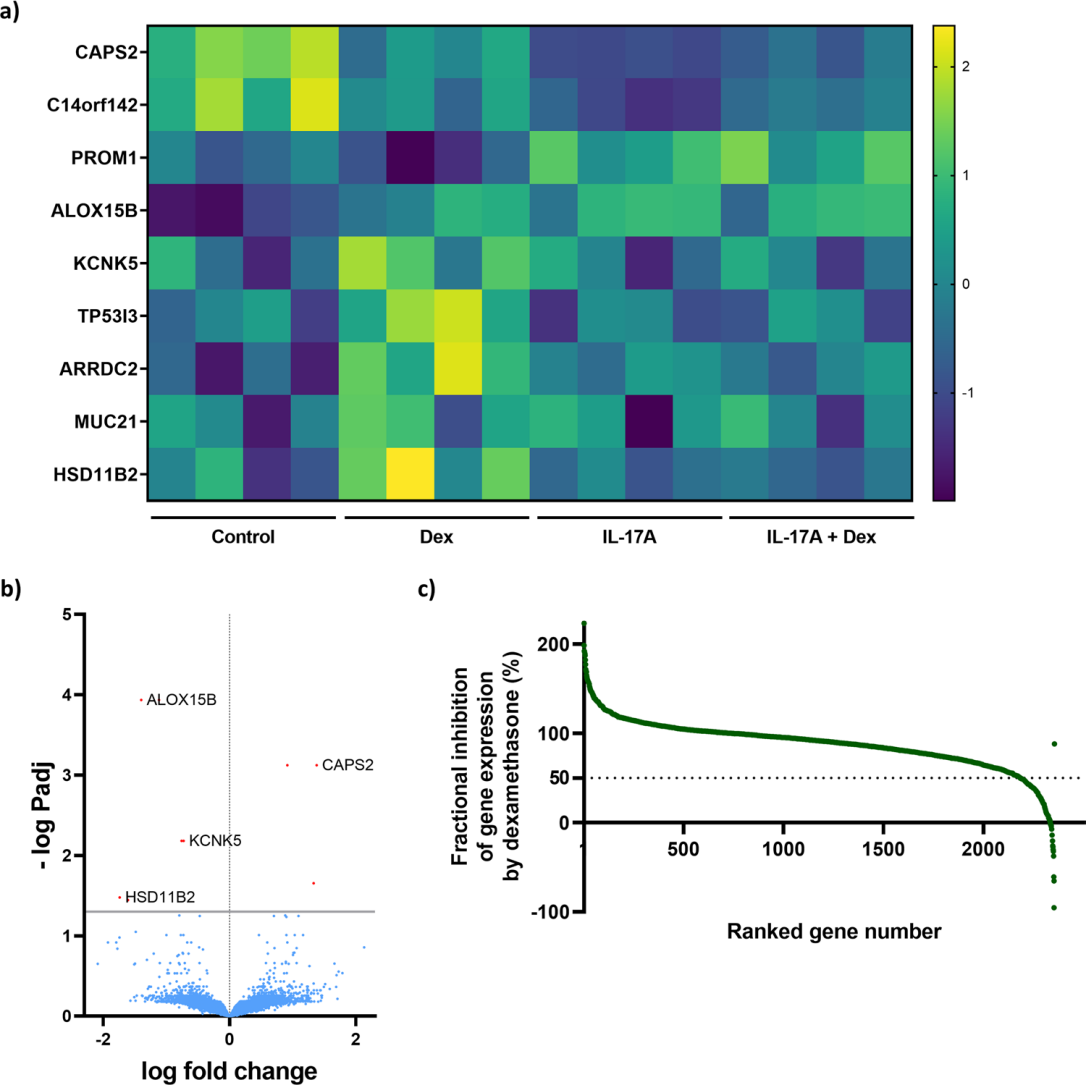
IL-17A and Dexamethasone (Dex) interaction in human airway epithelial cells (hAECs). hAECs were cultured and differentiated at an air liquid interface (ALI) and exposed to IL-17A (10 ng/ml) with or without dexamethasone (10 nM) for 14 days. Gene expression was determined by bulk RNA sequencing and differentially expressed genes were analyzed. 9 genes showed significantly interaction by IL-17A and dexamethasone and are presented in a heatmap, with each column in the groups representing an individual donor (**a**) and by a volcano plot (**b**). Genes that were differentially expressed by at least 1.5-fold response to IL-17A stimulation were included in the analysis and for these genes, the ability of dexamethasone to reverse this gene expression was assessed. The resulting score was normalized to 100%, yielding genes with a score of 100% as being completely dexamethasone resistant and genes with a score of 0% as being completely dexamethasone sensitive (**c**). N = 4 donors.

Since IL-17A regulated genes seemed to be insensitive to dexamethasone in the stimulated hAECs, we examined the IL-17A and dexamethasone interaction in differentiated hAECs in more detail and focused on genes for which there was a significant interaction effect between IL-17A and dexamethasone. There were nine genes that demonstrated significant interaction: *ARRDC2, ALOX15B, PROM1, CAPS2, KCNK5, TP53I3, C14orf142, HSD11B2,* and *MUC21* (*Padj* < 0.05) (Fig. 4a,b). Interestingly, these genes were also significantly regulated by dexamethasone alone, suggesting that the interaction is mainly affecting dexamethasone related genes (*Padj* < 0.05).

Surprised by the very strong repressive effects of IL-17A on dexamethasone signaling and the general lack of effect of dexamethasone on IL-17A signaling, we analyzed the effect of dexamethasone on IL-17A-related-genes in further detail. For the genes that were up/down-regulated by at least 1.5-fold in response to IL-17A stimulation the ability of dexamethasone to reverse this gene expression was assessed. The resulting score was normalized to 100%, yielding genes with a score of 100% as being completely dexamethasone resistant and genes with a score of 0% as being completely dexamethasone sensitive. Out of 2354 of these genes, only 170 (7.22%) genes showed strong inhibition by dexamethasone (≥ 50%), and the vast majority of 2184 genes (92.78%) were inhibited less than 50%) (Fig. 4c). This indicates that most of IL-17A-stimulated genes are less sensitive to dexamethasone, although dexamethasone sensitive genes do exist.

### Effect of IL-17A and dexamethasone on IL-17-induced inflammation

To elucidate the functional significance of dexamethasone sensitive and resistant IL-17A signaling in differentiated hAECs, we first examined their interactive effect on IL-17A inflammatory markers as well as neutrophilic inflammatory marker expressions, a known type of inflammation caused by high IL-17A stimulation in the airway^10,25,26^.

**Figure 5:**
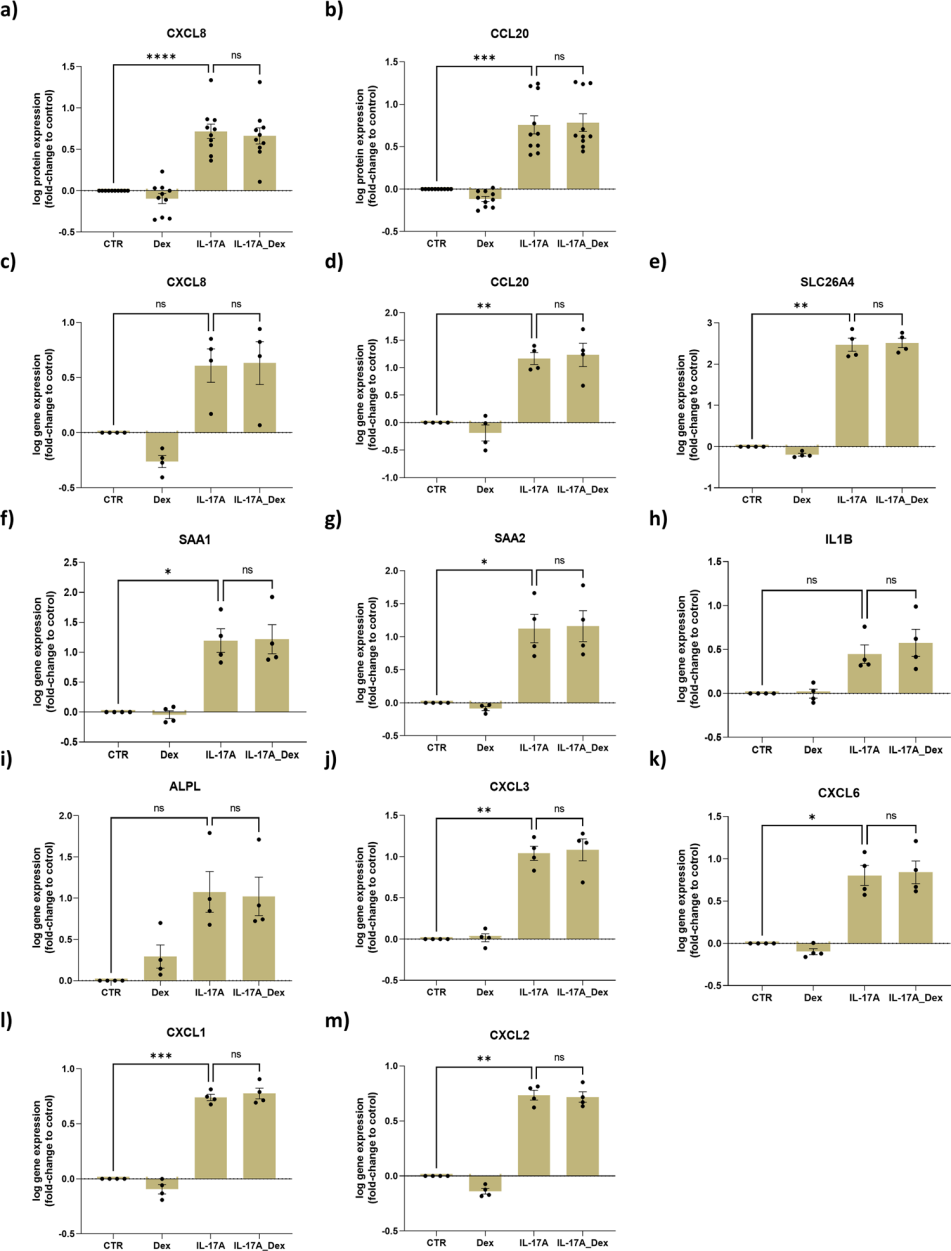
IL-17A stimulates IL-17A gene signature and inflammatory marker expression in human airway epithelial cells (hAECs), and these effects were not prevented by dexamethasone (Dex). hAECs were cultured and differentiated at an air liquid interface (ALI) and exposed to IL-17A (10 ng/ml) with or without dexamethasone (10 nM) for 14 days. CXCL8 and CCL20 protein release was measured by ELISA (**a**, **b**). *CXCL8, CCL20, SLC26A4, SAA1, SAA2, IL1β, ALPL, CXCL3, CXCL6, CXCL1*, and *CXCL2* gene expression was determined by total RNA sequencing and normalized gene counts were analyzed. Data represent means ± SEM. N = 10 donors for protein and N = 4 donors for gene expressions analysis. Statistical analyses were performed with paired one-way ANOVA and Tukey’s multiple comparison test, *p < 0.05, **p < 0.01, ***p < 0.001, ****p < 0.0001.

IL-17A significantly induced CXCL8 and CCL20 protein expression in basolateral medium (p < 0.0001 and p = 0.0003, respectively) (Fig. 5a,b). Both effects were completely resistant to dexamethasone (p < 0.05). Similarly, IL-17A effected gene expression of *CXCL8* (p = 0.0804), *CCL20* (p = 0.0053), *SLC26A4* (p = 0.0018), *SAA1* (p = 0.0271), *SAA2* (p = 0.0405), *IL1β* (p = 0.0690), *CXCL3* (p = 0.0038), *CXCL6* (p = 0.0196), *CXCL1* (p = 0.0004), *CXCL2* (p = 0.0015), and *ALPL* (p = 0.0650), and this effect was completely resistant to dexamethasone for all cytokines (Fig. 5c–m). This indicates that the IL-17A inflammatory pathway in hAECs is insensitive to corticosteroids.

### Effect of IL-17A and dexamethasone on epithelial mucus production

**Figure 6:**
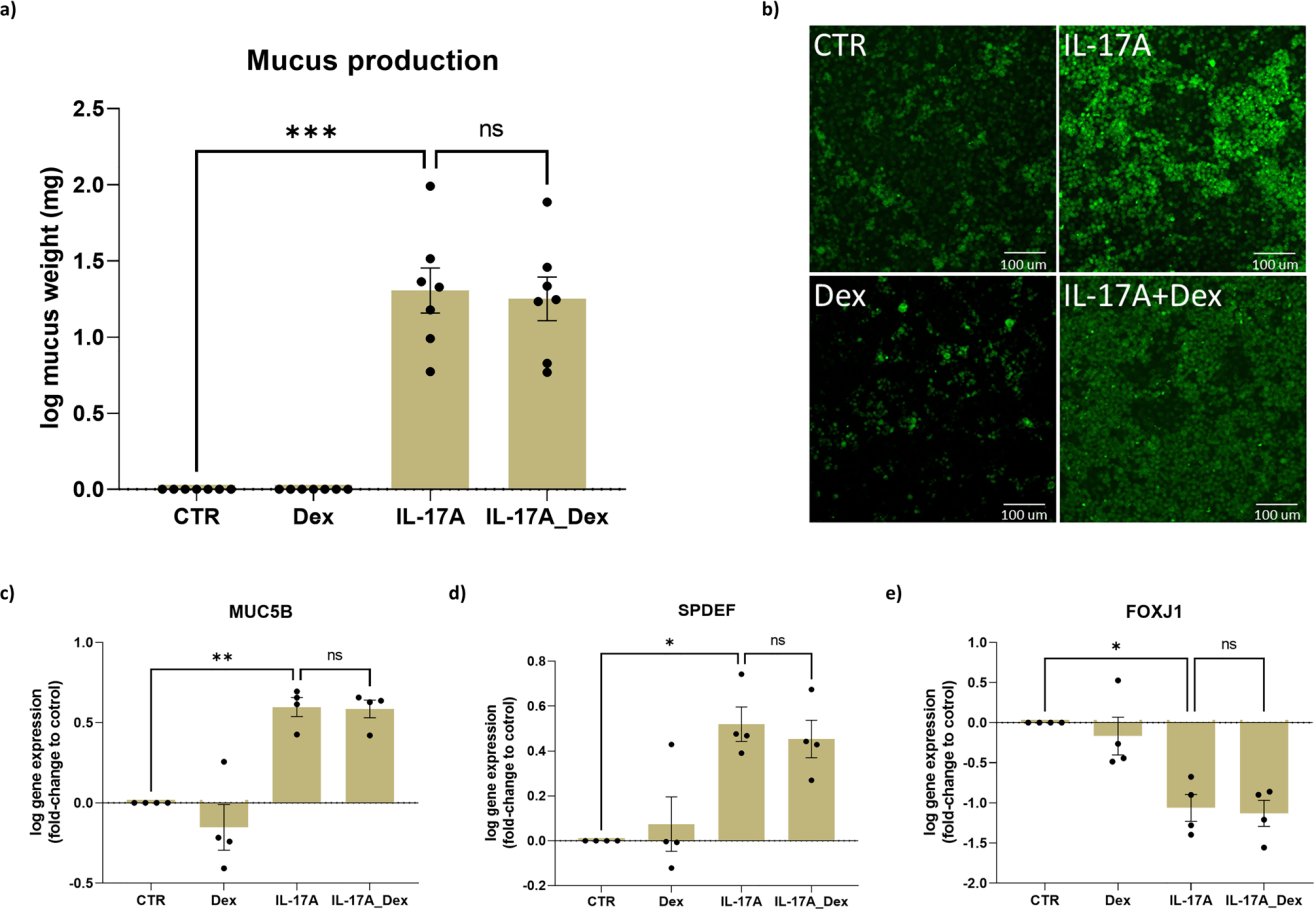
IL-17A induces goblet cell metaplasia and mucus production in human airway epithelial cells (hAECs), and these effects were not inhibited by dexamethasone (Dex). hAECs were cultured and differentiated at an air liquid interface (ALI) and exposed to IL-17A (10 ng/ml) with or without dexamethasone (10 nM) for 14 days. Mucus was collected and weighed at Day-11 (**a**). Representative images of ALI-cultured hAECs that were fluorescently labeled with MUC5AC (stained green (Alexa fluor 488); scale bar 100 µm; 20 X magnification) (**b**). Gene expression was determined by bulk RNA sequencing and normalized gene counts were analyzed (**c**–**e**). Data represent means ± SEM. N = 7 donors for mucus production measurement and N = 4 donors for gene expression analysis. Statistical analyses were performed with paired one-way ANOVA and Tukey’s multiple comparison test, *p < 0.05, **p < 0.01, ***p < 0.001.

Next, we investigated IL-17 functional interactions on epithelial mucus production. Mucus was collected and weighed every 2 days during the second week of differentiation. On day-11, IL-17A significantly increased mucus production and this was not inhibited by dexamethasone (Fig. 6a). Similar patterns of mucus production were also observed on day-8, day-13, and day-15 of differentiation period (data not shown). Consistent with this finding, epithelial goblet cell marker, MUC5AC, that was measured by immunofluorescence, were higher in IL-17A with and without dexamethasone groups (Fig. 6b). Other epithelial goblet cell markers *MUC5B* and *SPDEF* were also significantly increased at the gene expression level (p = 0.0064 and p = 0.0198, respectively), suggesting a shift towards goblet cell differentiation (Fig. 6c,d). In contrast, the ciliated cell marker *FOXJ1* was downregulated (p = 0.0236) (Fig. 6e). These effects were not prevented by dexamethasone. Together these results suggests that IL-17A stimulation leads to a shift towards mucus hypersecretory phenotype that is not prevented by dexamethasone.

### Effect of IL-17A and dexamethasone on airway epithelial integrity

**Figure 7:**
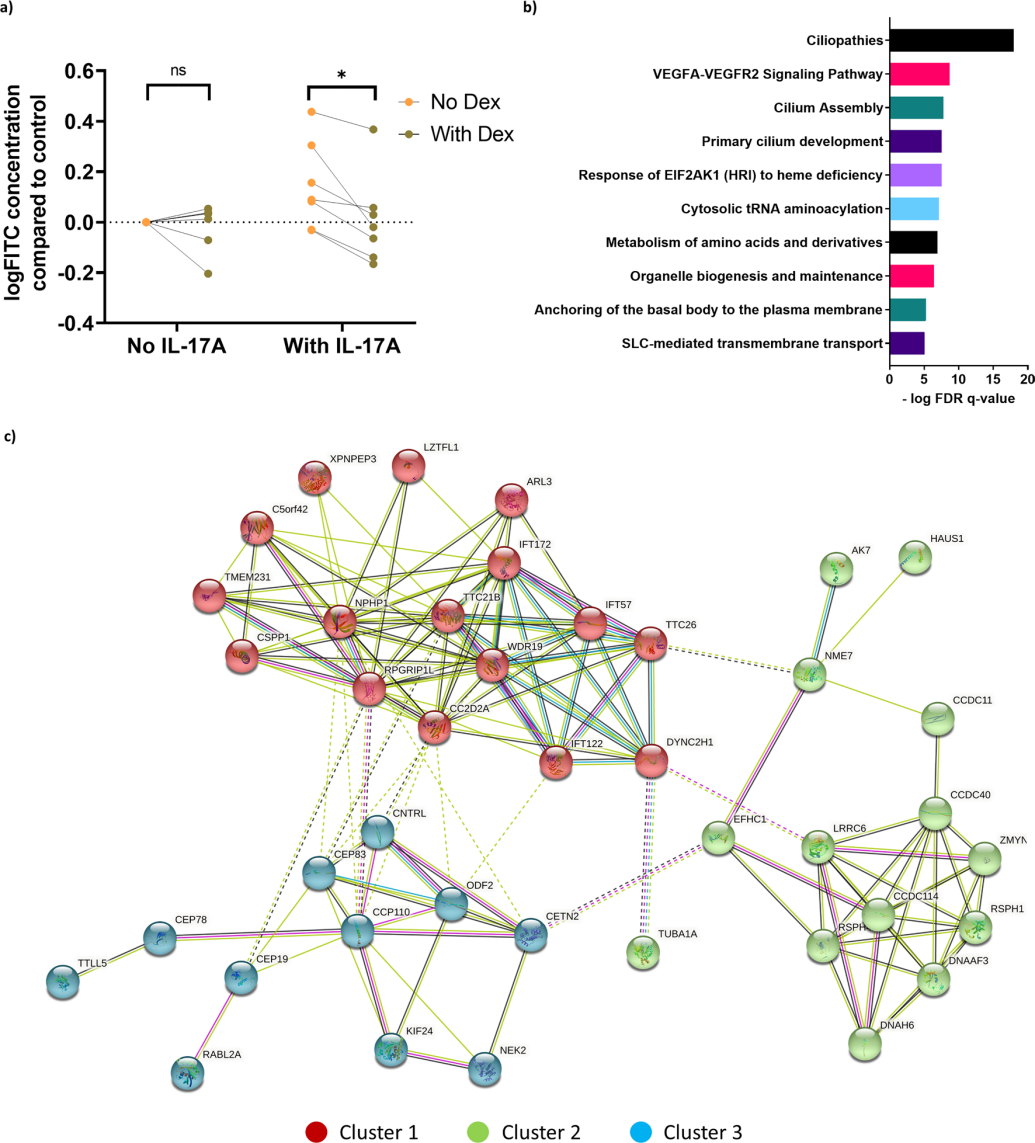
Dexamethasone (Dex) reverses IL-17A-induced epithelial barrier disruption in human airway epithelial cells (hAECs). hAECs were cultured and differentiated at an air liquid interface (ALI) and exposed to IL-17A (10 ng/ml) with or without dexamethasone (10 nM) for 14 days. Epithelial barrier function was assessed by permeability to fluorescein isothiocyanate-dextran (FITC-dextran) after 2 weeks incubation with IL-17A or dexamethasone of combination of both. FITC concentration was measured and calculated from fluorescence intensity. Data are from N = 7 donors (**a**). Gene expression was determined by bulk RNA sequencing and differentially expressed genes were analyzed. Statistically significant genes were used for pathway analysis (GSEA) and presented as top 10 most regulated pathways (**b**). String analysis (https://string-db.org/) was done to the genes that are related to cilia function and development (**c**). Data represent means ± SEM. N = 4 donors. Statistical analyses were performed with paired one-way ANOVA and Tukey’s multiple comparison test, *p < 0.05.

Next, we evaluated the epithelial integrity by FITC-dextran membrane permeability assay. Epithelial membrane integrity was measured after 2 weeks of differentiation. IL-17A stimulation caused epithelial membrane disruption, and the presence of dexamethasone significantly reversed this effect (Fig. 7a). To elucidate the corresponding genes that might be responsible for the effects on epithelial integrity, we reanalyzed the RNAseq data, with the inclusion of the FITC-dextran membrane permeability results into the analysis, to discover genes with a matching expression pattern. We found 749 genes that were significantly correlated with epithelial membrane integrity (*Padj* < 0.05), of which 337 and 412 genes were up- and down-regulated by IL-17A, respectively. Pathway analysis (GSEA) showed that 97 pathways were significantly regulated, with *Cilia function and development* as the most regulated pathway (FDR < 0.05) (Fig. 7b). This indicates that IL-17A-induced disruption of epithelial integrity and the reversal effect by dexamethasone are associated with *cilia function and development* regulation.

Furthermore, genes related with *Cilia function and development* pathway were analyzed with string analysis (Fig. 7c). There were 2 main clusters that were significantly involved: cluster 1 and cluster 2 (both with enrichment p-value < 1.0e − 16). Cluster 1 includes 16 genes (*LZTFL1, XPNPEP3, TMEM231, NPHP1, IFT172, C5orf42, CSPP1, RPGRIP1L, TTC21B, IFT122, CC2D2A, WDR19, TTC26, ARL3, IFT57, DYNCH21*) and cluster 2 includes 14 genes (*DNAH6, DNAAF3, ZMYND10, RSPH1, CCDC40, CCDC114, LRRC6, RSPH4A, CCDC11, EFHC1, NME7, TUBA1A, AK7, HAUS1*) (Fig. 7c; cluster 1 in red, cluster 2 in green). Previously, we showed that dexamethasone significantly regulated the *ciliopathies* pathway. This might explain the reversal effect of dexamethasone in IL-17A-induced barrier disruption here.

## Discussion

IL-17A regulates several airway epithelial functions in asthma and COPD, including stimulation of immune responses, airway remodeling, and mucus production. These effects have been reported as insensitive to corticosteroids^21,27–32^. In this study, we demonstrate that IL-17A induces function-specific corticosteroid insensitivity in differentiated primary human airway epithelial cells (hAECs) cultured at an air liquid interface (ALI). We demonstrate that IL-17A-induced inflammation and mucus production were not prevented by dexamethasone. Furthermore, we provide evidence that genes transactivated by dexamethasone, including *HSD11B2* and *FKBP5*, were dampened by the presence of IL-17A. In contrast, dexamethasone reversed IL17A induced hAEC epithelial barrier disruption, which was associated with gene expression changes related to cilia function and development.

Data from this study clearly indicates that IL-17A-induced inflammation is dexamethasone insensitive in primary hAECs. This is in line with previous studies showing that IL-17A-stimulated gene and cytokine expressions in primary hAECs were not inhibited by the presence of corticosteroids^21,32^. Furthermore, our data show that the expression of the IL-17A gene signature previously identified by Christenson et al.^10^ was not affected by the presence of dexamethasone. In contrast, the dexamethasone gene signature and corticosteroid transactivation genes that have been reported in literature, such as *HSD11B2* and *FKBP5*^23,24^, were downregulated by the addition of IL-17A. These data indicate that IL-17A might alter dexamethasone mechanisms of action and resulted in the lack of dexamethasone effect in primary hAECs.

Studies have indicated that corticosteroid insensitivity in airway epithelial is related to enhanced phosphoinositide-3-kinase (PI3K) pathway activity and a subsequent reduced HDAC2 activity, as well as elevated levels of GRβ expression, an inactive splice variant of the corticosteroid receptor, in response to IL-17A exposure^21,32^. Both mechanisms have been linked to corticosteroid insensitivity in asthma and COPD^33,34^. Moreover, IL-17-induced GRβ upregulation and subsequent corticosteroid insensitivity were also observed in peripheral blood mononuclear cells (PBMCs)^35^. Other IL-17A-related corticosteroid insensitivity mechanisms have also been reported in other cell types, such as increased MAP-ERK kinase 1 (MEK1) signaling in immune cells obtained from human bronchoalveolar lavage (BAL)^36^ and elevated colony stimulating factor 3 (CSF3), a key neutrophil survival cytokine, in human ASM cells^37^. In line with these findings, clinical data showed that COPD patients with a higher IL-17A gene signature have a lower response to ICS therapy with respect to the change in FEV1%-predicted-over-30-months^10^.

The notion that IL-17 is not only related to neutrophilic infiltration, but also a paracrine signaling cytokine in airway epithelial cells, has widened the perspective of the role of IL-17 in airway pathophysiology. It has been proposed that this direct role of IL-17 on airway epithelial cells might play a role in asthma and COPD pathophysiology. However, the mechanisms are still ill-defined^14^. In this study, ALI-cultured primary hAECs were used as a model to observe the direct role of IL-17A in airway epithelial cells. Moreover, since an ALI-cultured cells represent a pseudostratified mucociliary cell culture with various epithelial cells present, it is a more accurate comparison to the in vivo situation. Indeed, we observed that IL-17A stimulation upregulated the IL-17A gene signature, including *SLC26A4*, *SAA1*, *SAA2*, *CCL20*, *CXCL3*, *CXCL6*, *CXCL1*, and *CXCL2*, as was reported in a clinical study of patients with COPD^10^. Furthermore, our data showed that the *Neutrophil degranulation* pathway and neutrophil-activating chemokines, such as *CXCL8*, *CXCL3*, *CXCL6*, *CXCL1*, and *CXCL2*, *IL1β*, and *ALPL*, were upregulated upon stimulation with IL-17A in primary hAECs. Interestingly, IL-17A had direct effects on *Cilia function and development* as well as *Extracellular matrix (ECM) organization* pathways which were markedly regulated. Therefore, we propose that IL-17A effects on airway pathology might not only via inducing neutrophilic inflammation but also directly by altering airway epithelial cells.

Cilia function and development has a pivotal role in cell proliferation and differentiation^38–40^. More specifically, cilia loss is known to be related to aberrant cell differentiation^39^. Although studies on the role of IL-17A in cilia function and development of airway epithelial cells are limited, it was reported that IL-17A modulates ciliogenesis in keratinocytes, which is likely to driven by intraflagellar transport protein 88 (IFT88)^22^. Our data showed that IFT88 was significantly upregulated (*Padj* = 0.0033) by IL-17A stimulation. Moreover, pathways related to *Cilia function and development*, such as *Primary cilium development*, *Cilium assembly*, and *Ciliopathies*, were the most regulated pathways by IL-17A, involving 105 cilia-related-genes. Interestingly, the most regulated pathway by dexamethasone is also *Ciliopathies* that was contributed by 5 cilia-related-genes. We need to emphasize that our study did not include a detailed analysis of cilia function, or of ciliogenesis; this hypothesis was based on gene expression patterns only. Thus, it would be of interest to further investigate functional interaction of IL-17A and dexamethasone on ciliated cell development.

Another IL-17A-induced hAECs response that we observed is related to *Extracellular matrix organization*, including *Collagen formation*, *Degradation of extracellular matrix*, and *Collagen biosynthesis and modifying enzyme* pathways, involving 55 ECM-related-genes. In murine parenchymal epithelium, the expansion of Th17 cells through overactivation of STAT3 in T lymphocytes caused upregulation of matrix metalloproteinase-9 (MMP9) and ECM degradation that leads to airway epithelial remodeling^31^. This was shown to be an indirect consequence of neutrophil infiltration via CXCL5 and MIP-2 upregulation^31^. Although we did not observe significant regulation of MMP9 (*Padj* = 0.544) in our model, the gene expressions of other MMPs, such as MMP7, MMP13, MMP14, MMP15, and MMP17 were significantly regulated and contribute to *Extracellular matrix organization* pathway modulation. Our finding highlight IL-17A effects on airway epithelial ECM regulation which are independent from systemic inflammation. In fibroblasts, the direct effect of IL-17A on stimulating ECM production is via TGF-β activation in murine^29^ and human cells^41^. The involvement of NF-κB signaling and inhibition of JAK2, but not JAK1/3, in driving out this effect was also reported in human fibroblasts in vitro^42^. Therefore, elucidation of IL-17A molecular dynamics in regulating ECM in airway epithelium is an interesting topic to be studied further.

IL-17A is known to induce goblet cell hyperplasia and mucus production, which are prominent features of airway diseases, such as COPD, asthma, and cystic fibrosis^28–30,43^. Moreover, IL-17A is known to stimulate upregulation of SLC26A4, an anion exchange protein which is known to induce goblet cell hyperplasia in human lung mucoepidermoid carcinoma cells and murine airway epithelial cells^44–47^. Interestingly, SLC26A4 was found to be elevated in serum^48^, endobronchial biopsies^49^, and lung tissues of asthmatics^45^, and inversely associated with percentage of forced expiratory volume in 1 s (FEV_1_%), suggesting its role in airway disease^48^. Our data shows that SLC26A4 is the most differentially expressed gene in response to IL-17A, suggesting its significant role in driving goblet cell hyperplasia in our model. Furthermore, stimulation with IL-17A increased mucus production and the epithelial goblet cell gene markers, MUC5B and SPDEF, which were not inhibited by dexamethasone.

An important feature of airway remodeling in airway diseases is a change in epithelial barrier function. We observed reduced epithelial barrier integrity in IL-17A-stimulated hAECs as seen as increased FITC-dextran transmembrane permeability. An in vitro study in primary human nasal epithelial of chronic rhinosinusitis patients also reported similar results; a substantial epithelial barrier disruption, which was shown as elevated FITC-dextran permeability, loss of TEER, and reduced ZO-1 expression^50^. However, it is of great interest to investigate the underlying mechanism of IL-17A-induced airway epithelial barrier disruption, as it is yet to be unraveled. Studies that have been done in epidermal keratinocytes in vitro indicated that IL-17A impairs tight junction and epidermal barrier via downregulation of filaggrin and via genes involved in cell adhesion^51,52^. Thus, evaluation of changes in protein expressions related to airway epithelial tight junction and cellular adhesion that might resulted in barrier disruption, is the next interesting step forward in the future.

In conclusion, we demonstrated that IL-17A induces corticosteroid insensitivity in IL-17A-driven airway epithelial cell inflammatory cytokine responses and mucus production. In contrast, dexamethasone reverses IL-17A-stimulated epithelial barrier disruption. This indicates that IL-17A drives function-specific corticosteroid insensitivity.

## Methods

### Culture of human airway epithelial cells

Primary human airway epithelial cells (hAECs) were obtained from healthy lung transplant donors post-mortem, from residual tracheal and main stem bronchial tissue, within 1-8 h after a lung donation. Selection criteria for transplant donors are listed in the Eurotransplant guidelines and include the absence of primary lung disease, such as asthma and COPD, and no more than 20 pack years of smoking history. The material was collected in carbogenated Krebs–Henseleit-buffer (composition in mM: 117.5 NaCl, 5.6 KCl, 1.18 MgSO4, 2.5 CaCl2, 1.28 NaH2PO4, 25 NaHCO3 and 5.5 glucose). Epithelial cells were isolated by enzymatic digestion and cultured as described previously^53,54^. Cells were cultured in Keratinocyte Serum Free Medium (KSFM) medium with Bovine Pituitary Extract and EGF (Invitrogen, 17005042), supplemented with isoproterenol 1 µM (Sigma-aldrich, I6504). Experiments in submerged cells were conducted by exposing cells to 10 nM of Dexamethasone (Sigma-aldrich D4902), recombinant human IL-17A (R&D 7955-IL-025/CF) in three different concentrations (1, 10, and 100 ng/ml), or to combinations of IL-17A and Dexamethasone for 24 h.

For experiments in ALI cultured cells, cells from passage 1–2 were grown submerged on collagen-coated Transwell inserts until confluence, after which they were cultured at an air–liquid interface (ALI) and differentiated for 2 weeks in B/D medium (1:1), which is a mixture of DMEM [Gibco] and bronchial epithelial growth medium (BEGM) [Lonza], supplemented with 0.4% [w/v] bovine pituitary extract [BPE], 0.5 ng/ml epidermal growth factor [EGF], 5 µg/ml insulin, 10 µg/ml transferrin, 1 µM hydrocortisone, 6.5 ng/ml T3, 0.5 µg/ml epinephrine [all from Lonza], 15 ng/ml retinoic acid [Sigma chemical] 1.5 µg/ml bovine serum albumin [Sigma chemical] 0.5 mM sodium pyruvate [Gibco], 20 U/ml penicillin and 20 µg/ml streptomycin [Gibco]^53^. Cells were exposed to 10 nM of Dexamethasone (Sigma-aldrich D4902), 10 ng/ml of recombinant human IL-17A (R&D 7955-IL-025/CF), or the combination of IL-17A and Dexamethasone that was added to the basal medium during two weeks of differentiation. ALI cultures were maintained for 14 days at 37 °C in a humidified atmosphere of 5% CO_2_. Medium and stimuli were refreshed three times per week. The apical side of the epithelial cells was washed with phosphate buffer saline (PBS) at the same time. After 2 weeks, cells were collected for RNA analysis or used for barrier function experiments, mucus was collected for volume measurement, and basal medium was collected for cytokine analysis.

### RNA analysis

Total RNA was isolated using the RNA purification kit NucleoSpin RNA (Macherey–Nagel 740955, Germany) according to the manufacturer’s guide. Cells were lysed by adding a mixture of buffer RA1 and dithiothreitol (Sigma-Aldrich, 3483-12-3). The yield of the isolated RNA was then measured using the NanoDrop 1000 spectrophotometer. Samples were analyzed by RT-PCR or RNA sequencing (RNA-seq).

For RT-PCR analysis, equal amounts of total RNA were reverse transcribed into cDNA. cDNA was generated with cDNA synthesis kit (Promega) using M-MLV reverse transcriptase (Promega M1701) in Doppio thermal cycler (VWR, 732-2551). RT-PCR analysis were performed in Quantstudio 7 flex Real-Time PCR system (Thermo Fisher Scientific, 4485701). RT-PCR was performed with denaturation at 94 °C for 30 s, annealing at 59 °C for 30 s and extension at 72 °C for 30 s for 45 cycles followed by 10 min at 72 °C. RT-PCR data were analyzed using the comparative cycle threshold (Ct: amplification cycle number) method. The amount of target gene was normalized to the endogenous reference gene RPL13a and SDHA.

RNA-seq was conducted using the Illumina NovaSeq 6000 sequencer by GenomeScan (https://www.genomescan.nl/). The procedure included data quality control, adapter trimming, alignment of short reads and feature counting. Library preparation was checked by calculating ribosomal (and globin) content. Checks for possible sample and barcode contamination were performed and a set of standard quality metrics for the raw dataset was determined using quality control tools (FstQC v0.34 and FastQA). Prior to alignment, the reads were trimmed for adapter sequences using Trimmomatic v0.30. To align the reads of each sample, the human reference GRCh37.75 was used. Data was analyzed with DESEQ2 in R studio v1.4.1106.

Z-scores were calculated as follows:

$$z-score=\mathrm{log}\left(\frac{(x-\upmu )}{\upsigma }\right)$$

where: x is the data point, µ is the mean, σ is the standard deviation.

FDR q-values were assessed using the Gene Set Enrichment Analysis webtool, Broad Institute, UC San Diego (https://www.gsea-msigdb.org/gsea/index.jsp).

### ELISA

Basal media were used for ELISA quantification of human CCL20 and CXCL8 following the instructions of the commercial kit (DM3A00 and D8000C, respectively, R&D systems).

### Barrier function

Barrier function was assessed by permeability to 4000 Da fluorescein isothiocyanate-dextran (FITC-dextran, Sigma-Aldrich). Cells were washed once with HBSS (Thermo Fisher) and 500 µl of 1 mg/ml FITC-dextran in HBSS was added to the apical chamber. Cells were incubated for 6 h at 37 °C in a humidified atmosphere of 5% CO2, after which 100 µl HBSS with tracer was collected from the basolateral chamber in duplicate. Permeability to FITC-dextran was evaluated through measuring the fluorescence intensity at 490/520 nm using a fluorescence plate reader (Synergy H1, Biotek).

### Mucus analysis

Mucus was collected from the apical side of the ALI-cultured cells and weighed every 2 days during the second week of differentiation.

### Immunofluorescence

For mucin 5AC (MUC5AC) protein visualization, ALI-cultured hAECs cells on top of the insert’s membranes were fixed in acetone:methanol 1:1 (volume/volume) for 15 min at − 20 °C. After fixation, the inserts were washed with 1X phosphate buffer saline (PBS) 3 times and blocked with 3% H_2_O_2_ for 30 min in room temperature. The inserts were further washed and incubated with the primary antibody mouse anti-MUC5AC (1:100; Fisher Scientific Epredia MS145P) in 1% BSA and 2% donkey serum for 1 h in room temperature. Next, the inserts were washed and incubated with the secondary antibody donkey anti-mouse Alexa Fluor 488 (1:200, Invitrogen A21202) for 30 min in the dark in room temperature. The inserts then were cut, inverted, and transferred to glass slides with 2 drops of the mounting medium containing DAPI (Abcam 104139). The slides were kept at 4 °C. Confocal images were acquired using Leica SP8 microscope at 20× magnificantion.

### Statistical analysis

All data were presented as mean ± SEM unless indicated otherwise. Data was analyzed for statistical significance using one-way or two-way ANOVA. The padj-value indicating statistically significant differences between the mean values are defined as follows: *p < 0.05, **p < 0.01, ***p < 0.001, ****p < 0.0001. Statistical analyses were performed with Graphpad Prism 9.2.0 software.

## Data Availability

The datasets generated during and/or analysed during the current study are available from the corresponding author on reasonable request.
